# Supplementary material for: Delayed healthcare seeking and associated factors for common childhood illnesses among caregivers with under-five children who visited Yem special woreda public health facilities, Southwest Ethiopia, 2023
Source: PLoS One. 2024 Oct 17;19(10):e0297707. doi: 10.1371/journal.pone.0297707 (PMC11486390; doi:10.1371/journal.pone.0297707)
Supplement: Attachment — Submitted filename: Response to Reviewers.docx [file pone.0297707.s001.docx]

**Response to Reviewers**

First, I would like to appreciate the reviewers and editors for adding a substantial input as suggestion and comments to the manuscript so that the paper would meet the required standards of scientific article.

Below The authors provided their responses to the raised comments.

Regarding Journal requirements:

1. The manuscript is modified to meet the font and styling requirements of the journal.
2. Regarding ethics, the participants were required to agree verbally after the data collectors read/provided all the information about the study. Each questionnaire sheet has an information sheet and consent form pages which was intended to be told to the caregivers before starting the interview. At the end of consent form there is two options: ‘***agree to continue’*** and ‘***do not agree’***. If the caregivers verbally agree to continue to be interviewed, the data collector will mark to the tick box in front of ‘***agree to continue’***. Verbal informed consent was taken because in rural areas people fear to sign consent even though they agree to participate in the study. This was approved by Jimma University IRB.
3. All additional data and information are made available separately along with the manuscript.

**Response to Reviewer #1**

1. Operational definitions revised.
2. The perception of severity about their child’s illness was measured by asking the caregivers to rate the severity of the current illness by their own judgement. They were asked whether the illness was mild, moderate and severe according to their personal perception.
3. Line 274, table 3: the inconsistency was checked. In row 2, participants may have more than one source of information, therefore the sum up may not give the same number as sample size, greater than sample size.
4. **Reviewer comment**: “Visited a health facility (n=130) Yes 90 69.2 No 40 30.8 Previous visit help for today's visit. It’s not clear for me because your study was institution based so ….”

**Author response:** This is about the previous experience of the caregivers. It asked about whether the caregivers seek healthcare for the last illness during the last 6months. Therefore, this question was asked for caregivers whose children encountered illness in the last 6 months.

5. **Comment:** on Decision maker figure I have a doubt with the context of our country…

**Authors response**: The caregiver was asked “Who decided to take the child for medical treatment?” This may not represent the overall decision maker among the family.

6. **Comment:** Exaggerated figures on alcohol, smoking:

**Authors response:** The numbers are increased because the caregivers were asked to drinking alcohol in the last 12 months including local drinks which have alcohol such as *tella*, *araqe, katikala…* these increases the response rate to the question because it is traditionally common to drink this local alcohol-containing drinks. The same is true for smoking as most people smoke a locally produced nicotine containing substance called “*gaya”*

7. **Comment:** Major issue is about analysis totally it needs revision. For example, variable\Distance to the health facility (within walking/ is not associated in binary…COR 1.39(.84-2.28), Information about early health care seeking, Knowledge of danger signs.

**Authors Response: Yes,** these variables are not associated in the bivariate analysis. However, we use p-value <0.25 to filter variables for multivariable analysis. Therefore, the variables presented in the regression table (Table 5) are variable that have p-value <0.25, not necessarily have significant association with the outcome variable.

8. **Comment**: your discussion is shallow…please elaborate more

**Authors response:** Discussion is modified.

**Response to Reviewer #2**

**General Comments**

1. Regarding the representativeness of the sample, the authors modified the study settings to Yem special woreda. Hence, the manuscript is modified as “*Delayed Healthcare Seeking and Associated Factors for Common Childhood Illnesses among Caregivers with Under-Five children who visited Yem special woreda public health facilities, Southwest Ethiopia, 2023.”* The whole document is revised accordingly.
2. Regarding the adequacy of the design to address the objectives of the study, the authors believe that the objectives stipulated in the study can be addressed by the design. However, the detailed aspects of the problem can still be explored by the qualitative study, which the authors recommend for future research.
3. Concerning the sample size adequacy, the authors have applied all the assumptions required for sample size calculations. All considerations were applied to get maximum sample size. Sample size was initially estimated by single population proportions formula for the first objective (magnitude of delayed healthcare seeking) and double population proportion formula for the second objective (factors affecting delayed healthcare seeking) by considering all the assumptions for each approach. Then the maximum sample size was obtained (single population proportion formula gave the maximum sample size). Therefore, as long as all assumptions are fulfilled, we believe that the sample size is adequate for generalization and cross-sectional study.
4. Concerning the statistical analysis applied, the authors tried our best to clearly describe the statistical analysis applied in the study. See Page 2 Line 44 and ….

***Specific comments***

*Introduction*

1. **Comment:** “*This study does not sound novel to me. Since there are so many studies on related topics worldwide, including Ethiopia. Therefore, the authors need to try harder to clarify the motivation and contributions of this study*.”

**Authors response:** Majority the studies conducted on health seeking behavior of caregivers overlooking the time at which they seek, however the current study focuses on the proportion caregivers who delayed care seeking. Moreover, the rationale for conducting this study and its contributions is described in the revised manuscript**. See *Page 5 line 128-138*.**

2. **Comment:** *The introduction needs to be well organized. Similar paragraphs should be synthesized.*

**Authors response:** introduction reorganized. Paragraphs synthesized. See P3L64-66, P3L72-81, P4L90-104

3. **Comment:** Why does southwest Ethiopia matter in this study?

**Authors response**: The study setting is modified to Yem special woreda. This is area with underprivileged health systems and majority rural community. It is area where one of the authors work and caregivers are observed to bring their children to medical attention late. This interests the author to study the problem in the area.

4. **Comment:** The scientific background and rationale for the investigation is not clearly addressed, which you raised as a gap should be clearly stated.

**Authors response:** Addressed according to comment given. See ***P5 Lines 117-128***

5. **Comment:** Your introduction is not comprehensive; it did not cover all variables assessed in your study example knowledge of caregivers, behavior, previous experiences **Authors response**: the issue is Addressed. See ***page 4 lines 110-116.***
6. **Comment**: I’m not clear with your objective. Is it to assess delays in healthcare-seeking and associated factors for common childhood illnesses among caregivers of under-five children in Southwestern Ethiopia or Yem special woreda public health facilities?

**Authors response**: The objective is corrected as Yem special woreda public health facilities.

*Methods and materials*

1. The study setting is revised as “Yem Special Woreda public health facilities”. The whole document is modified accordingly.
2. **Comment:** “Unpublished data should be acknowledged.”

**Authors response**: Revised accordingly.

3. Inclusion and exclusion criteria: Revised according to comment given.
4. Study design is inserted separately as an independent subheading as per the comment*.*
5. Regarding sampling procedure: systematic sampling procedure is applied because of the nature of the flow with which the study participants came to the Under-5 OPD. The caregivers visit the health worker by order of their coming to health center. It is not possible to generate a sampling frame, therefore simple random sampling is not possible. The other sampling techniques like purposive sampling prohibits the equal chance of the participants to be selected in the study. Therefore, the authors believe that SS techniques is the appropriate technique.
6. Measurement and operational definition are separated. Subheading “measurement” is inserted.
7. Is the data collection tool standardized? How?

***Authors Response:*** The data collection tool was developed after reviewing relevant literature. The authors couldn’t get standardized tools. However, the validity and reliability of the questionnaire was checked. The validity was checked by the subject matter experts. The reliability of the test was checked by Cronbach alpha, & it was 0.804.

8. The actual value of Hosmer-and-Lemeshow value is inserted. See page P9 L222.
9. Method of variable selection for your multivariable analysis inserted as suggested.
10. How have you tried to control possible confounding and biases?

YES, the authors controlled the possible confounding and biases by using multivariable analysis.

11. ***Comment:*** “Page 8, line 209-11, rephrase the sentence and remove the redundant statement.

**Author’s response:** The statement is rephrased as “Statistical association between dependent and independent variables was determined at P-value <0.05. Variables that are statistically significant were identified as factors for delay in health care seeking.”

12. **Comment:** “Page 8, line 217-19, authors mentioned pretest and validity test was carried out. Could you supplement them with results? What modification or revision did you do for your tool?”

***Authors response:*** The result of the pretest and modifications done is included in the revised manuscript.

13. **Comment:** Page 8, line 223, “The collected data were reviewed and checked for completeness everyday by the supervisors and principal investigator.” Could you elaborate who is principal investigator and who are supervisors?

**Authors response:** the statement is revised as “The collected data were reviewed and checked for completeness everyday by the supervisors and weekly by principal investigator.” In addition, principal investigator is the one responsible the whole study starting from the conception to the development of the research proposal/protocol including the data collection tool, while supervisor is someone recruited so that he/she facilitates and monitor the field work/data collection. Two supervisors were recruited to follow the data collection progress at each facility.

14. Data processing and analysis subheading is revised. The Hosmer-and-Lemeshow value is added, method of variable selection for your multivariable analysis is added.
15. Ethical considerations revised and re-paraphrased according to the comments.

Results:

1. **Comment:** Did the authors used the standard variable categorization? Especially for ages, number of family,...

**Authors Response:** The intention behind age classification is to see delay of health seeking in infants less than one year and those children older than one year. Moreover, this categorization was done based on previous literatures.

2. **Comment:** Page 13, Caregivers’ previous experiences-related factors out of 130 children visited a health facility only 40 of them cured of illness?

**Authors response:** The item is corrected as “If not visited health facility, how was the child treated of illness (n=40)”. The question was about health seeking practices for the previous illnesses in the last 6 months. See **page 14.**

3. **Comment**: I believe that some findings are out of scope. If it is necessary, it should be included on the background and objective of the study.

**Authors response**: The variables are included in the introduction section of the manuscript. See ***page 4 lines 110-116.***

4. **Comment:** If assessing substance use is necessary, it should be holistic. For example, you should include frequency, duration, amount, …

**Authors Response:** The tool asks about alcohol consumption in the last 12 months. In addition to that the frequency of their consumption was addressed in the questionnaire. The amount of alcohol an individual consumed cannot be measured in this study as the standard unit of measurement is not feasible to measure in the rural communities.

5. **Comment**: Approximate or round the numbers to the nearest while interpreting odds ratio. For instance, instead of saying 1.99 times, say 2 times…

**Author’s response**: Revised according to the suggestion given. See page ***16 lines 327-339.***

6. Discussion is modified according to comment given from reviewers.
7. Conclusion is added to the manuscript.
8. Limitation of the study is added to the revised manuscript.
9. Recommendations section added to the manuscript. See page 21 lines 436-440

Thank you.
