# Supplementary material for: Delayed healthcare seeking and associated factors for common childhood illnesses among caregivers with under-five children who visited Yem special woreda public health facilities, Southwest Ethiopia, 2023
Source: PLoS One. 2024 Oct 17;19(10):e0297707. doi: 10.1371/journal.pone.0297707 (PMC11486390; doi:10.1371/journal.pone.0297707)
Supplement: Attachment — Submitted filename: Reviewer-Comments.docx [file pone.0297707.s002.docx]

# **Reviewer**

**Manuscript Number:** PONE-D-24-00553
**Title:** Delayed Healthcare Seeking and Associated Factors for Common Childhood Illnesses among Caregivers with Under-Five Children in Southwestern Ethiopia, 2023.

**General comment:**

The study was done on an important public health issue as Ethiopia is still in a plight of pervasive under-five child mortality. Thus, the manuscript can be considered for publication as it stands now.

However, I need some clarification, and if it is possible, with a correction suggestion on some idea or sentence in the manuscript that makes me confused and is not clear.

**Specific comments**

1. You were reporting both good and bad knowledge of danger signs. What is your base, to say, good or poor? Knowledge of danger signs for a particular study participant or caregiver means that how do you measure their knowledge and what is your evidence?
2. Delayed healthcare seeking is categorized as “yes” or "no." What is your base to categorize as “yes” or “no” because you are not clearly operationalized at the section of operational definition, just you are only giving a definition of what is "delay in healthcare-seeking" means?”
3. I try to see in detail your methodology section, so I feel that there is a missed sub-section, which is “study variable.” Under this section, you must clearly state your independent (explanatory) variable and dependent (outcome) variable in this section. Off course I understand that you are trying to put some independent and dependent variables under the Operational Definitions section, but your study variable must be put separately under the sub-section of the study variable. Then you can operationalize these variables, which makes them confused when someone reads your paper with their appropriate definition and how you measured them.
4. What are your inclusion and exclusion criteria? You missed this one also under methodology its good putting in separate subsection of inclusion and exclusion criteria.
5. Please add a citation or reference for your line 207 whey 0.25; why not 0.2? Some studies also use 0.2 as a cutoff point. Wherever, put your evidence for your cut point if the study recommends or uses a cut point of 25%.
6. Data quality control: What about Cronbach's alpha value to approve wither your data consistent or not?
7. Data processing, analysis: What about multicollinearity and correlation of your data to approve wither your data normal or not? And
8. Finally, I suggest carefully looking at the grammar error and all text formatting, like spacing and indentation, removing unnecessary spacing between paragraphs, and also checking the PLOS one manuscript submission format. I detect such a problem in your document.

In general, this paper is written well. Information and knowledge reflected in this study is valuable for policy development for countries and organizations and stakeholder groups who are working on child care, and it is also a good input for developing an inclusive starting plan for family health.
