# Supplementary material for: Delayed healthcare seeking and associated factors for common childhood illnesses among caregivers with under-five children who visited Yem special woreda public health facilities, Southwest Ethiopia, 2023
Source: PLoS One. 2024 Oct 17;19(10):e0297707. doi: 10.1371/journal.pone.0297707 (PMC11486390; doi:10.1371/journal.pone.0297707)
Supplement: Attachment — Submitted filename: Rebuttal Letter.docx [file pone.0297707.s003.docx]

**Rebuttal Letter**

The authors are very grateful to the editor and reviewers for giving their precious time to critically review the manuscript and providing vital comments and suggestions to make our document more insightful.

Below is the response to the concerns raised by the reviewers

**Letter to editors**

1. Borderlines removed from table 1, 2, 3, 4 and 5
2. References are edited.

**Response to Reviewer #3**

1. Line 72-81 paragraph is edited and rewritten. ***See Line 72-82***
2. Population, sample size estimation and Sampling procedure edited. Diagrammatic presentation of sampling procedure inserted. ***See line150-174***.
3. The study employed systematic random sampling technique. Since all the public health facilities in the Yem special woreda (the study area) were included in the study, Multistage sampling technique was not applied in the study. Therefore, the authors believe there is no variation in estimation requiring design effect.
4. Recommendation: Recommendation section is removed as per the journals guidelines and merged into discussion section. The suggested recommendations are edited and aligned with findings of the study. ***See line 449-458***

**Response to reviewer #4**

1. Knowledge of danger signs was measured based on caregivers’ ability to spontaneously mention WHO danger signs. There is **no** **universally agreed up on** base to classify caregivers’ knowledge of danger signs. However, researchers use different cut-off points to classify knowledge. Based on this the authors classify knowledge based on the number of danger signs they are able to mention. Therefore, those caregivers who listed less than three (3) danger signs were classified to have poor knowledge of danger signs, while those who listed three and above danger signs are classified as having good knowledge of danger signs.
2. **Reviewer’s comment:** “*Delayed healthcare seeking is categorized as “yes” or "no." What is your base to categorize as “yes” or “no” because you are not clearly operationalized at the section of operational definition, just you are only giving a definition of what is "delay in healthcare-seeking" means?”*

**Author’s response:** Operational definition for delayed healthcare seeking and healthcare seeking not delayed separately defined in the revised manuscript. **See line 211-214**

3. Study variables subsection is separately inserted. ***See line 175-189***
4. Eligibility criteria inserted as a separate subsection. **See line 154-159**
5. Comment addressed as the reviewer suggested. Reference inserted. **See line 244-245**

The use of a higher p-value threshold (e.g., 0.25) for variable selection reflects the idea that the bivariate analysis alone does not account for the effects of other variables. A less strict threshold helps to avoid discarding variables that could still have a meaningful impact when adjusted for other predictors in the multivariable model. Different statistical books suggest p-value as high as 0.25 for variable selection. Few are mentioned below:

In the step where variables are being screened for possible inclusion in the multivariable model, we recommend screening variables using a p-value of 0.25 for inclusion in the model.”
 (Hosmer & Lemeshow, 2000, p. 95).

“We suggest selecting variables with p-values of 0.25 or less in the bivariate analysis for inclusion in the multivariable model.”
(Bursac et al., 2008). **Reference**: Bursac, Z., Gauss, C. H., Williams, D. K., & Hosmer, D. W. (2008). Purposeful selection of variables in logistic regression. Source Code for Biology and Medicine, 3(1), 17. doi:10.1186/1751-0473-3-17

6. **Reviewers comment:** Data quality control: What about Cronbach's alpha value to approve wither your data consistent or not?

**Authors response:** The value of the Cronbach’s alpha was inserted. It is within acceptable range showing the internal consistency of the tool to measure the variables. ***see line 262***

7. Reviewers comment: “Data processing, analysis: What about multicollinearity and correlation of your data to approve wither your data normal or not? And”

Authors response: Multicollinearity checked, and value inserted in the main manuscript. ***See line 243-246***

8. Editorial checking: Grammar, editorial and punctuations are checked and corrected to the best of the author’s ability.
